# Direct writing of gold nanostructures with an electron beam: On the way to pure nanostructures by combining optimized deposition with oxygen-plasma treatment

**DOI:** 10.3762/bjnano.8.253

**Published:** 2017-11-29

**Authors:** Domagoj Belić, Mostafa M Shawrav, Emmerich Bertagnolli, Heinz D Wanzenboeck

**Affiliations:** 1 Institute of Solid State Electronics, TU Wien, Floragasse 7, 1040 Vienna, Austria; 2 University of Liverpool, Department of Chemistry, Crown Street, Liverpool L69 7ZD, United Kingdom; 3 Institute of Sensors & Actuator System, TU Wien, Gusshausstrasse 27–29, 1040 Vienna, Austria

**Keywords:** FEBID, gold nanostructures, oxygen plasma, postdeposition purification

## Abstract

This work presents a highly effective approach for the chemical purification of directly written 2D and 3D gold nanostructures suitable for plasmonics, biomolecule immobilisation, and nanoelectronics. Gold nano- and microstructures can be fabricated by one-step direct-write lithography process using focused electron beam induced deposition (FEBID). Typically, as-deposited gold nanostructures suffer from a low Au content and unacceptably high carbon contamination. We show that the undesirable carbon contamination can be diminished using a two-step process – a combination of optimized deposition followed by appropriate postdeposition cleaning. Starting from the common metal-organic precursor Me_2_-Au-tfac, it is demonstrated that the Au content in pristine FEBID nanostructures can be increased from 30 atom % to as much as 72 atom %, depending on the sustained electron beam dose. As a second step, oxygen-plasma treatment is established to further enhance the Au content in the structures, while preserving their morphology to a high degree. This two-step process represents a simple, feasible and high-throughput method for direct writing of purer gold nanostructures that can enable their future use for demanding applications.

## Introduction

Focused electron beam induced deposition (FEBID) is an additive direct-write method for making complex 2D and 3D nanostructures [1–5] with an ultimate resolution of less than 1 nm [6–7]. It is frequently referred to as "3D nanoprinting" because of its capabilities that are similar to the increasingly popular 3D printers that operate on the micrometer scale. FEBID has been only recently widely utilized [8–10] due to the growing number of modern scanning electron microscopes (SEMs) which can be combined with focused ion beam (FIB) systems that are equipped with gas injection systems by default. Rather than being merely characterization instruments, such SEMs are now becoming important fabrication tools for the production of nanoscale structures and devices in magnetics [11–15], superconductors [16], and nanoelectronics [17–19]. The use of FEBID processing as a nanosurgery method for novel nanomaterials, such as graphene, is particularly appealing [20–24]. Hence, any advancement made in the production [25–28] or in situ purification [29] of FEBID materials attracts great attention.

Among all FEBID materials, Au is of special interest because its superior properties that make it highly suitable for use in micro- and nanotechnology, as well as in nanomedicine [30]. Many applications require precise positioning of Au structures on the nanoscale, e.g., for fabrication of interconnects [31–32] and field emission tips [33] or in direct writing of plasmonic and photonic nanostructures and devices [4,34–40]. We recently utilized FEBID of Au to directly write metal-oxide-semiconductor capacitors [41] and to deposit Au nanocatalyst templates for Si nanowire syntheses [42].

The main issue which is hindering the widespread use of FEBID is the low Au content in the produced nanostructures: when using the common, commercially available metal-organic FEBID Au precursors, such as dimethylgold(III)-acetylacetonate (Me_2_-Au-acac), dimethylgold(III)-trifluoroacetylacetonate (Me_2_-Au-tfac) or dimethylgold(III)-hexafluoroacetylacetonate (Me_2_-Au-hfac), deposits typically contain less than 30 atom % of Au [33,37]. This value was only slightly increased under deposition in reactive environments [43–44]. Logically, it would seem that the best way to avoid carbon contamination is to use a carbon-free precursor. Indeed, FEBID experiments using the PF_3_AuCl precursors yielded almost pure Au structures [32], but this precursor was eventually abandoned due to its poor stability. Other gold complexes are under consideration [45–46], but have not been adopted in practice due to significant experimental deficits. Therefore, the low Au content in FEBID structures obtained from commonly used commercial precursors remains an outstanding issue. For that reason, this study focuses on the optimization of deposition mechanism using a commercially available metal-organic gold precursor and explores oxygen-plasma treatment as a potentially viable large-scale postdeposition purification method for 2D and 3D nanostructures.

Over the last few years several methods to purify FEBID deposits have been presented. For instance, in the case of tungsten, it was found that just by applying a smart writing strategy one could produce single-crystal nanowires [47] whereas the use of a supersonic carrier gas can also yield nanostructures of a high purity [48]. For cobalt, a combination of heat, H_2_ exposure and electron irradiation improved the metal content in the deposits [49]. Similarly, in the case of platinum, several purification approaches have been studied, including laser assisted purification [50–51], a sequential EBID process and additional postdeposition electron irradiation in the presence of H_2_O or O_2_ [52–56]. The mechanisms behind these processes point to fundamental differences between molecular oxygen and water as oxidative species [57].

Similar purification methods have also been investigated for gold, but different surface mechanisms prevented the direct application of the methods used for Pt purification to Au purification. These differences between the platinum precursor MeCpPtMe_3_ and the acetylacetonate-based gold precursors Me_2_-Au-acac, Me_2_-Au-tfac, and Me_2_-Au-hfac become evident by the fact that atomic layer deposition is feasible for platinum [58–59], but has proved very challenging for gold [60–61]. While the encouraging results from Pt purification can provide inspiration, it is still necessary to develop new procedures for the purification of FEBID gold.

Roughly, all these procedures that strive for purer FEBID gold can be categorized into two groups – ones that deal with (in situ) optimization of deposition processes and the others that deal with various postdeposition (ex situ) treatments to further improve the purity of the deposits. Prime examples of the latter group include heat treatment in a reactive atmosphere of air [62] or oxygen [63], but these methods are compromised by an (often significant) loss of structural fidelity. Similarly, room temperature electron beam postirradiation in the presence of O_2_ [64] or H_2_O [4] has been used to purify FEBID gold in a second step, but this method is limited to smaller structures and areas that can be efficiently processed by a scanning electron beam. As a possible solution to this issue, oxygen-plasma treatment, has been explored, yielding promising results [65–66]. Nevertheless, the ultimate goal is direct deposition of high-purity gold nanostructures, removing (or minimizing) the need for any postdeposition purification. Recently, notable advances have been made in this line of work. For example, in situ purification during deposition using simultaneous flows of both O_2_ and precursor yielded up to a 1:10 C/Au ratio [67]. This one-step process requires a special custom-designed dual nozzle-in-nozzle setup that is currently not readily available to the majority of FEBID users. Our recent work on simultaneous injection of gold precursor and water vapour during deposition outlines an experimentally simpler approach that seems to be very effective for realizing pristine nanostructures of high purity [68]. However, injection of water directly into the microscope chamber may not always be feasible or preferred, so conceiving an even simpler deposition strategy is desired. The simplest possible method would be to optimize the deposition parameters, making use of conventional equipment and a standard precursor. The ultrahigh vacuum/surface science approach to studying the effect of electron beam irradiation on nanometer thin films of a common Me_2_-Au-acac precursor from Wnuk et al. [69–70] represents a valuable starting point for optimization of FEBID Au nanoarchitectures. Similar to their findings, our previous study on FEBID Au nanopillars (NPs) revealed that unusually large Au crystals could be found at the bases of the NPs, which was attributed to in situ focused electron beam induced curing (FEBIC) during deposition [65]. That investigation explored a wide range of experimental FEBID parameters in a systematic manner, in order to yield a recipe for deposition of pristine 3D nanostructures with desired structural and compositional properties. With a systematic experimental approach to FEBID with metal carbonyl precursors it was possible to obtain outstanding pure pristine Fe and Co nanostructures [71]. Although the decomposition reaction of pyrolytic precursors is essentially different to the Au chelate complex used as precursor in this study, we have used an analogous systematic approach to improve the metal content of the deposit.

The carbon contamination, which mainly originates from the imperfect chemical decomposition of the precursor, is a strong limitation for possible usage of FEBID nanomaterials in optical, magnetic, electronic or information storage devices. It is therefore essential to improve their purity by optimizing the chemical reaction pathway. This experimental study focuses on the advancement of the material purity of FEBID Au by combining optimized (in situ) deposition with (ex situ) oxidative plasma treatment. We revisit the commonly used metal-organic Me_2_-Au-tfac precursor and show that by smartly setting the deposition parameters, one can achieve pristine FEBID nanostructures of exceptionally high Au content without the use of additional reactive species within the SEM. In a second step, we employ chemical purification by oxidation. We demonstrate that such postdeposition treatment of Au nanostructures can further boost the Au content without compromising their structural stability, indicating that the procedure could be suitable for larger and more complex FEBID architectures.

## Results and Discussion

**Figure 1:**
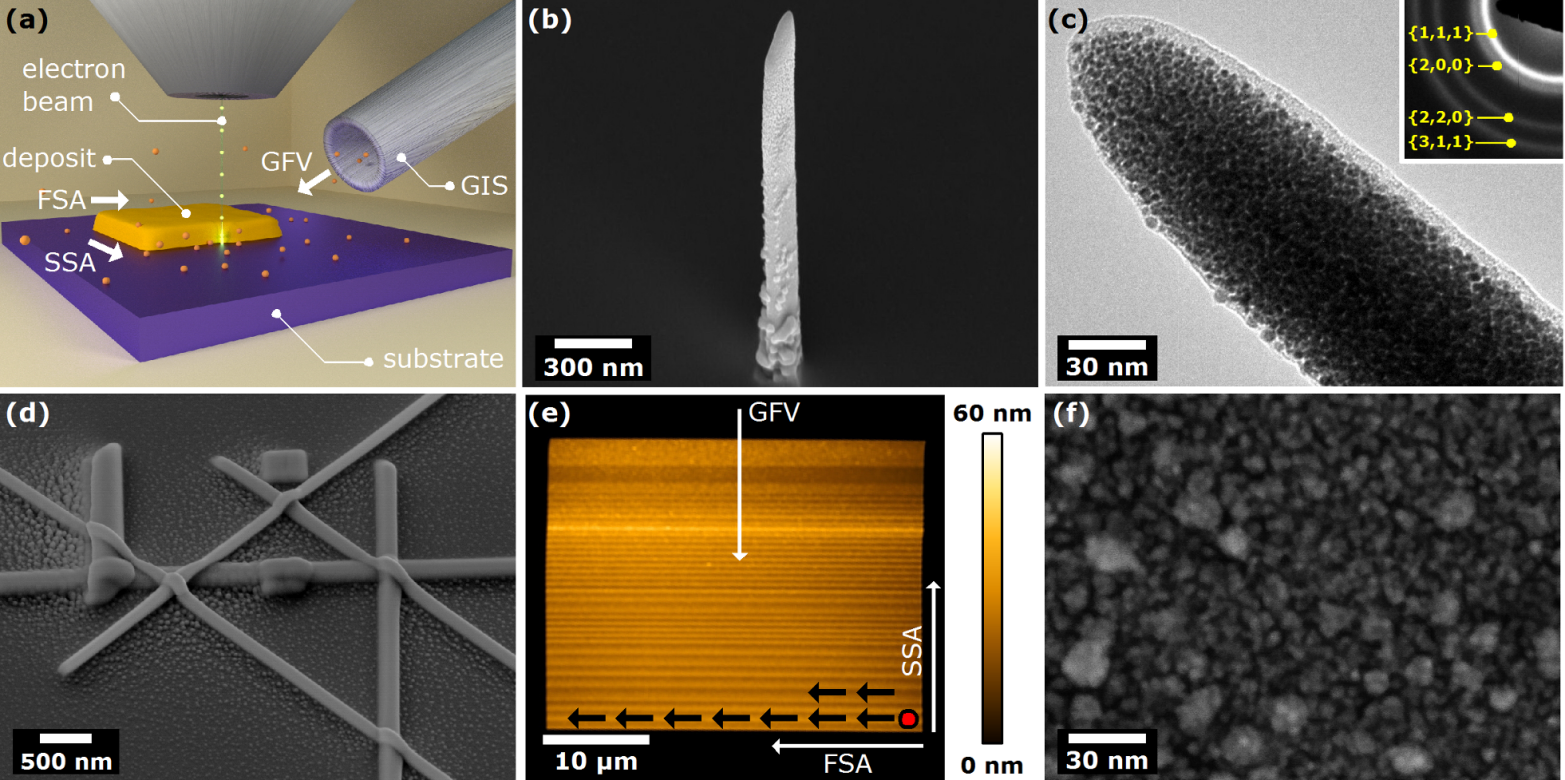
(a) Schematics of the FEBID experimental set-up. Note that the direction of electron beam movement, i.e., fast scanning axis (FSA) and slow scanning axis (SSA), can be adjusted relative to the gas flux vector (GFV). (b) SEM image (45° tilt) of a Au nanopillar (NP) fabricated by FEBID. (c) TEM image of a FEBID Au NP, with the corresponding electron diffraction pattern given in the inset, confirming the polycrystalline nature of the NP. (d) SEM image (45° tilt) showing a group of various Au nanostructures produced by FEBID. (e) AFM topographic image of a relatively large planar Au structure made by FEBID. Electron beam scanning strategy was adjusted so that FSA is perpendicular to GFV, whereas SSA was opposite to GFV. Note the height variation of the deposit in direction of SSA. (f) HAADF-STEM image (top view) of a section of a planar FEBID Au structure, deposited on a thin silicon nitride membrane.

We deposited 2D and 3D Au nanostructures as a proof-of-principle because such structures are desirable for devices for bio-nanotechnology, nanoelectronics, plasmonics and photonics. The nanostructures were prepared using FEBID, as schematically depicted in Figure 1a. In brief, precursor molecules (dimethylgold(III)-trifluoroacetylacetonate, chemical formula C_7_H_10_AuF_3_O_2_) were introduced into the high-vacuum chamber of a custom-modified SEM via home-built gas injection system (GIS) [42], in such a way that a nozzle was brought into close vicinity (≈200 μm) of the intended deposition spot on a prepared p-type Si substrate. When a beam of primary electrons impacts on the surface, secondary electrons are generated by inelastic interactions, having energies suitable for breaking the bonds of the precursor molecules adsorbed to the substrate. Consequently, a metal-containing material was deposited with a considerable amount of incorporated carbonaceous species mostly due to the partial break-up of precursor molecules. By varying the experimental parameters one can alter the deposition process and hence change the properties of deposited material, such as its shape, structure, and elemental composition. We note that no etching by the F found in the precursor was observed.

Firstly, we investigated the FEBID of vertical Au NPs as model structures to elucidate how the variation of the experimental parameters influenced the metal content in the NPs. The electron beam impinging on a single spot on the substrate allowed us to produce free-standing NPs of a relatively large height-to-width aspect ratio (up to 50), measuring several micrometers in height while only having ≈100 nm in diameter (Figure 1b). Transmission electron microscopy (TEM) revealed the polycrystalline nature of the NPs having 3–8 nm crystallites embedded in an amorphous matrix (Figure 1c), which seems to be a common feature of Au NPs produced by FEBID in non-reactive environments [38,43]. The corresponding electron diffraction pattern given in the inset of Figure 1c is consistent with polycrystalline Au, revealing interplanar distances of (2.4 ± 0.1) Å, (2.1 ± 0.1) Å, (1.4 ± 0.1) Å, and (1.2 ± 0.1) Å that correspond to the spacing between {1,1,1}, {2,0,0}, {2,2,0}, and {3,1,1} planes in the face centered cubic (fcc) Au lattice, respectively.

Apart from the deposition of NPs, we used FEBID to directly write horizontal Au nanowires (NWs), NW networks, and planar structures (Figure 1d,e). In the latter case, the deposited structures had an area measured in hundreds of square micrometers, making them suitable for applications in electronics [41]. However, FEBID of such large structures is very challenging and requires very stable experimental conditions over a long time. For instance, the planar structure presented in Figure 1e had been deposited for 8.5 h while the working pressure during the introduction of precursor gas was maintained at 1.2 × 10^−5^ mbar. In a calibration experiment with nitrogen gas a chamber pressure of 1.2 × 10^−5^ mbar corresponded to a flux of 0.11 sccm. During such a long experiment the precursor supply varied up to 15 rel %, as derived from the chamber pressure readout. The precursor supply was manually readjusted via the temperature setting of the precursor reservoir.

The depositions were performed with a single pass scan with the e-beam writing line-by-line from top left to bottom right, as is indicated in Figure 1e. In prolonged experiments a small beam drift present during deposition resulted in rectangular structures instead of the original square pattern. The uneven (wrinkled) surface of planar FEBID structures can be the result of an inhomogeneous beam drift or of precursor flux variation, which we deem more likely.

To maximize the deposition rate, i.e., to reduce the total deposition time, the electron beam parameters – acceleration voltage, i.e., high tension (HT), current (*I*), dwell time (DT), and pixel-to-pixel spacing (PPS) – had to be properly adjusted. It was experimentally found that using HT = 3 kV and *I* = 1.0 nA (i.e., 60 μm condenser aperture) resulted in the fastest growth of both 2D and 3D nanostructures, for a given DT and/or PPS. We note here that the maximum deposition rate usually does not lead to the purest deposit (in fact, quite the opposite was observed). Once the deposition rate was optimized and the samples were produced, high-angle annular dark field scanning transmission electron microscopy (HAADF-STEM) revealed that the crystallites in the planar deposits were considerably larger than those in NPs (compare Figure 1f to Figure 1c). Although both samples were produced using the same HT and *I* values, the electron dose that the material sustained during deposition was approximately two times higher in the case of planar structure (2.3 × 10^−7^ C·μm^−2^ vs ≈1 × 10^-7^ C·μm^−2^). It was the first clear evidence that a change of electron beam parameters could significantly alter the inner structure of FEBID Au deposits, deserving greater attention. In the next sections of this work we present the main results of our comprehensive study of the influence of electron beam parameters on the properties of Au nanostructures fabricated by FEBID.

### Au nanopillars

Generally, the full experimental parameter space of a typical FEBID system is multidimensional – it encompasses electron beam parameters (such as acceleration voltage, i.e., high tension, electron beam current, electron dose, i.e., deposition time), precursor parameters (such as precursor type, flux, and possibly addition of other gases), and substrate parameters (such as substrate type and temperature). To reduce the level of complexity, here we restrict the full parameter space to a subspace spanned by the axes of high tension, electron beam current, and deposition time.

The deposition of Au NPs was carried out at 4 different HT values: 1 kV, 3 kV, 5 kV, and 10 kV, using a 60 μm condenser aperture, which provided a current of 1.0 nA, as was set by the condenser lens and measured by a Faraday cup placed inside the SEM chamber. Additionally, for the fixed HT of 3 kV, the electron beam current was varied by using the 30 μm, 60 μm, and 120 μm condenser apertures. For every HT and current, we set the deposition time to 20 s, 40 s, and 60 s and produced at least 6 NPs using identical experimental conditions. In total we deposited over 120 samples and conducted a broad SEM and EDX study to elucidate the effect of electron beam parameters on the deposition rate, morphology, and elemental composition of NPs. For the sake of clarity we here concentrate on the results obtained for 60 s deposition at the current of 1.0 nA, with varying HT values. These samples are chosen as they provide the clearest picture of the behaviour generally observed across all Au NP samples.

All NPs fabricated under the same experimental conditions appeared indistinguishable in terms of both the morphology (as evidenced by SEM imaging) and chemical composition (as evidenced by SEM EDX linescans); see Figure S1, Supporting Information File 1. Therefore, the results shown here are highly representative for the whole group of NPs produced under the same conditions. The top section of the pillar slightly below (50–150 nm) the tip was analyzed, as this section was more similar in diameter to the bottom part. The chemical analysis of the topmost tip of the nanopillar is plagued by the small self-absorption of the carbon K-line in the tip region and the depth-dependence of the absorption in tapered tip geometries. We would like to point out that a thickness-dependent error for the given results still exists, so the measured data might slightly differ from the actual purity levels.

**Figure 2:**
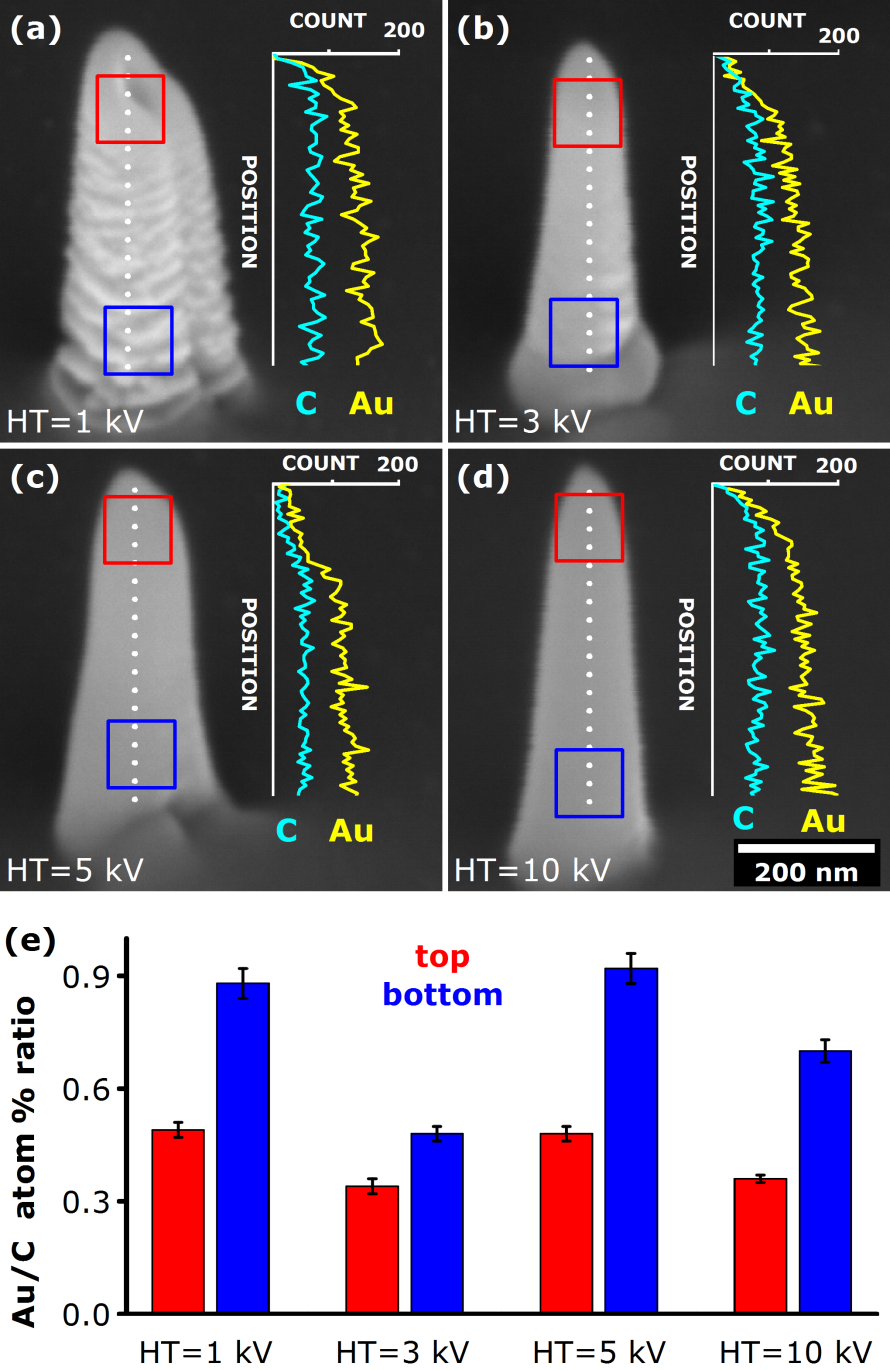
(a)–(d) SEM images (45° tilt) and the corresponding EDX line profiles taken along the height of the Au nanopillars (NPs), as indicated by the dotted line (to the scale). These vertical NPs were deposited for 60 s using an electron beam current of 1 nA for various acceleration voltage values, as indicated in the images. By definition, the top section relates to the top quarter of the NP, whilst the bottom section relates to the bottom quarter, as indicated by the red and blue squares, respectively. The scale bar applies to all SEM images in this Figure. (e) Elemental composition in the top and bottom sections of samples presented in (a–d), expressed in terms of Au/C atom % ratio. The EDX measurements were performed on 100 × 100 nm^2^ areas with an acquisition period of 60 s. For detailed EDX data see Table S1 in Supporting Information File 1.

Commonly, for the same deposition time and current (aperture), the height of the NPs was approximately equal regardless of the HT used, although the appearance of the NPs changed from more rugged and wider, produced using the lowest HT value of 1 kV, to smoother and thinner, deposited at higher HT values (Figure 2), in line with previous observations for W [72]. SEM EDX studies showed that the NPs are in reality mostly made up of C, having a smaller fraction of Au present in the deposits (see Figure S2, Supporting Information File 1). A small O fraction was also detected (<10 atom %), but it remains unclear whether it partially originates from decomposed precursor molecules (C_7_H_10_AuF_3_O_2_) or partially from the atmospheric O_2_ and H_2_O that had been adsorbed to the NPs during a short transfer of the samples under ambient conditions from the deposition system to the analytical SEM. The amount of F in the NPs was found to be below the detection limit of EDX measurements, which is a bit surprising considering the fact that the precursor molecules contained more F than Au or O atoms.

Detailed linescan EDX investigations of produced samples revealed a compositional gradient in literally every NP that was sufficiently tall for multiple probing: as a rule of thumb, the gold fraction gradually increased from the top to the bottom of the NPs, by as much as 57% (relative) in the case of 10 keV beam (from ≈24 atom % at the top to ≈38 atom % at the bottom; see Table S1, Supporting Information File 1). At the same time, the carbon fraction showed the opposite trend, notably decreasing towards the bottom of NPs. It is worth noting that the O content did not exhibit any evident variation between the top and bottom parts of NPs, which indicates the dominant presence of uniformly adsorbed O atoms. It was commonly observed that the Au gradients in NPs deposited for shorter periods were not as evident as in NPs that were deposited for longer periods (Figure S2, Supporting Information File 1). These results were obtained repeatedly and were independent of the direction of EDX scanning and the relative position of the sample to the EDX detector. We note that very similar composition values and gradients were found by STEM EDX performed on long straight (uniform diameter) sections of NPs deposited on silicon nitride TEM grids in our previous study [65]. More accurate compositional quantification could probably be achieved by performing atom probe tomography or thickness-corrected STEM electron energy loss spectroscopy (EELS).

### Planar Au nanostructures

The main goal here was to elucidate the effects of each experimental parameter on the properties of planar nanostructures; in particular, we wanted to see which parameter had the strongest influence on the elemental composition of the deposited structures, in order to find the experimental recipe that will maximize the Au content in pristine planar FEBID structures. As such, this investigation represents a major step towards fabrication of exceptionally pure, patterned planar Au nanostructures by giving crucial pointers for their practical straightforward realization.

**Figure 3:**
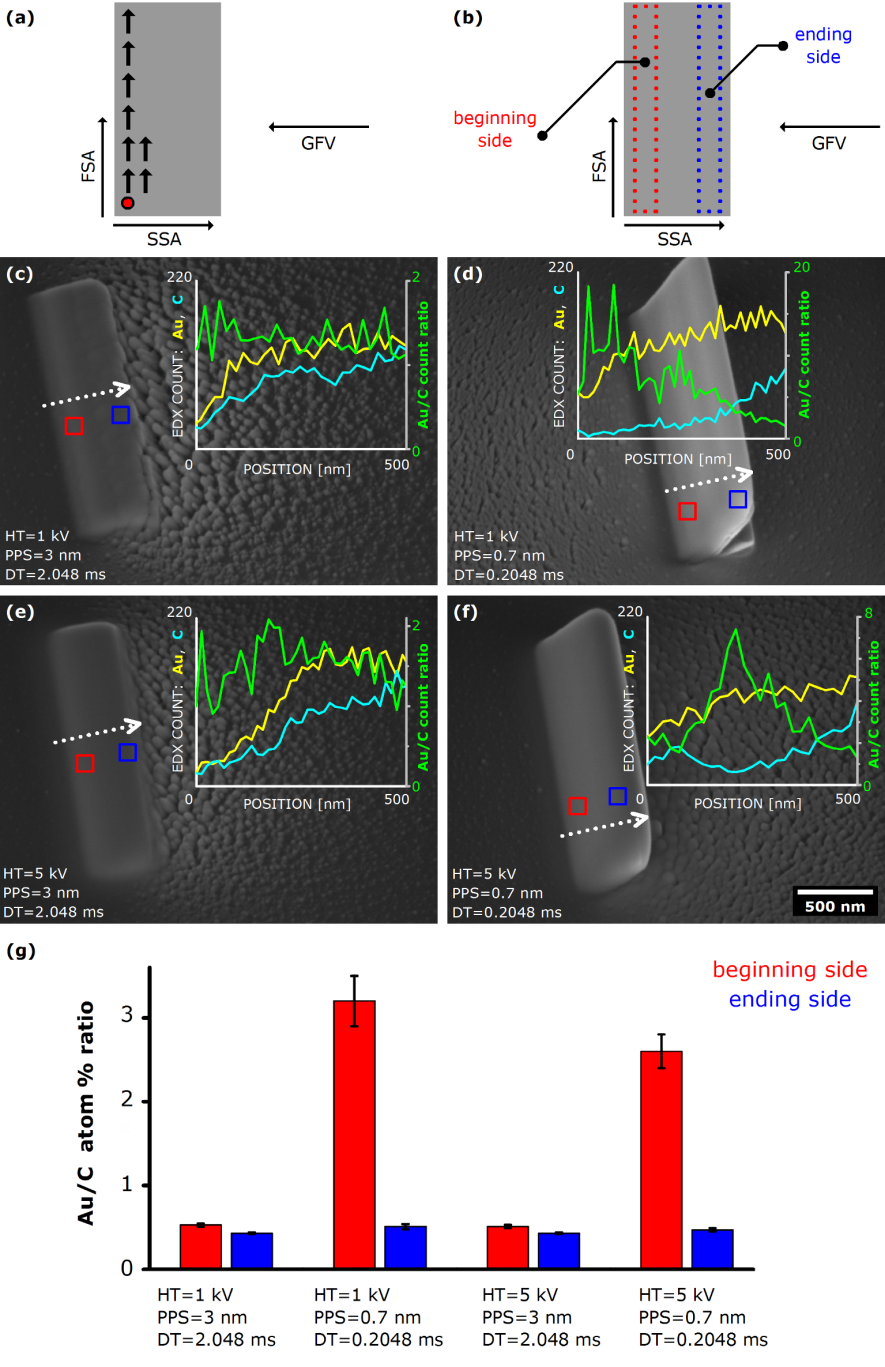
(a) Schematics of the scanning strategy used in FEBID of planar structures. (b) Definition of “beginning” and “end” of the structures, with respect to the scanning strategy. Note that both areas are ≈50 nm away from the respective edges of the planar structure. (c–f) SEM images (tilted view) of planar Au structures measuring 2 × 0.5 μm^2^ in area, deposited using various experimental conditions. Plots containing EDX line profiles are given in the insets. Note the change of scale for Au/C count ratio among the images. The scale bar indicated in (f) applies to all SEM images in this figure. (g) Elemental composition of the planar structure presented in (c–f), as determined by EDX measurements performed on 100 × 100 nm^2^ areas for 60 s, on the “beginning” and “end” of the deposition, marked with a red and blue square, respectively. Note a pronounced Au content on the “beginning” side in the samples produced using a higher electron beam dose. For detailed EDX data see Table S2 in Supporting Information File 1.

As an archetype for all planar FEBID experiments in this study, we deposited rectangular structures measuring 2 × 0.5 μm^2^ in area, using a single pass, with PPS set to either 3 nm or 0.7 nm, and DT set to either 0.2048 ms or 2.0480 ms. These specific values are selected on the basis of results of an initial set of experiments performed on smaller 0.5 × 0.5 μm^2^ structures which suggested that sufficiently small PPS and/or sufficiently long DT lead to an increase of the Au content in the deposits. However, setting too small PPS and too long DT drastically increased the total deposition time to a few hours, even for a relatively small planar nanostructure having an area of a few square micrometers. Thus the PPS and DT values had to be chosen wisely in order to keep the whole study experimentally feasible. The overall strategy was to firstly produce a planar structure by setting PPS to 3 nm and DT to 0.2048 ms, which represents the smallest electron dose per unit area. In the second step, keeping PPS at 3 nm, DT was increased to 2.048 ms. In the final step, PPS was decreased to 0.7 nm, with DT set back to 0.2048 ms. By following such a procedure, the total electron dose was varied relatively as approximately 1:10:18, for a given electron beam current. The procedure was performed for HT values of 1 kV, 3 kV, 5 kV, and 10 kV for the fixed current of 1.0 nA (condenser aperture of 60 µm). In total more than 80 samples were deposited and subsequently analysed. The main results of the study are presented in Figure 3; for the sake of clarity and brevity, we only show results for a limited set of samples that still provide clear insight into a general trend.

It is clear that more material was deposited when the electron dose was higher (compare Figure 3c,e to Figure 3d,f). The growth rate of the samples produced at HT = 1 kV and HT = 5 kV was around 0.43 μm^3^/μC. The volume of the deposited material was found to be linearly dependent of the electron dose which, in combination with relatively long dwell times and high currents used in these experiments, indicates that we were most likely working in the mass transport regime [73]. There were notable asymmetric, disjointed deposits (“halo”) present around the main planar nanostructure, with more material deposited in the SSA direction, clearly indicating a shadowing effect. This likely occurred due to scattering of primary electrons on already deposited material, which resulted with favoured generation of secondary electrons in front of the deposit (as seen from the deposition direction). On the other hand, one might speculate that the body of already deposited main structure may have obstructed the supply of adsorbed precursor molecules and/or secondary electrons to the back side. These observations are in agreement with the previous reports on FEBID of Cu [74] and Pt [75]. In spite of the “halo”, the main structure remained fairly well defined for all HT values used. Interestingly, in the case of maximum electron dose, the planar structures at one point start to lift up and deposit under an angle relative to the substrate plane – a 2D analogue of 1D lines from [74]. According to our experimental results, this “disconnection electron beam dose” lies between 2.3 × 10^−7^ C·μm^−2^ and 4.2 × 10^−7^ C·μm^−2^ for electron beam energy ranging from 1 keV to 10 keV.

A comprehensive EDX investigation of the planar structures followed and detailed line EDX profiles were acquired, bearing in mind that EDX profiling inevitably induces some unwanted C deposition from the residual hydrocarbons present in the analytical SEM chamber. To minimize this effect which may have consequently lead to misleading results, the chamber containing the samples was always pumped overnight prior to SEM characterization. The chemical composition was probed on two sides of the structures, defined as “deposition beginning” side and “deposition end” side, with respect to the electron beam/deposition advancement. It was found that the chemical composition was almost uniform across the whole 2 × 0.5 μm^2^ structure for the samples produced with the lowest electron beam dose (see Figure 3c,e), regardless of the acceleration voltage used during the deposition. Notably, the composition of these planar structures was quite similar to the composition of the top sections of vertical Au NPs.

In the case of deposition with higher electron beam exposure doses (Figure 3d,e), the results clearly reveal that the composition is not consistent across the whole area of the structures: although the lateral line profiles (i.e., along FSA) showed no significant compositional variation in that direction, the profiles taken in the SSA direction exhibited large differences from one side of the deposit to the other. Evidently the “deposition beginning” side was systematically richer in Au than the “deposition end” side. For instance, in the case of the sample produced by setting HT = 1 kV, PPS = 0.7 nm, and DT = 0.2048 ms (Figure 3c), the Au/C atom % ratio was nearly 7 times higher on the “deposition beginning” side than on the “deposition end” side. The same behaviour of Au and C content was usually observed for other highest electron dose samples with no exception, albeit at smaller differences between the opposite sides of planar structures. This behaviour in planar Au nanostructures seems fundamentally equal to the one observed earlier in the study of Au NPs, where the bottom sections of NPs were consistently Au-richer than the top sections. In particular, the long dwell time “planar” nanostructures that lift-off from the substrate exhibit compositional profiles very similar to those seen in the nanopillars. This indicates that the optimization of pixel spacing may enable fabrication of planar structures of a high gold content, which is of interest in the context of nanotech applications. These findings strongly indicate that the elemental composition of FEBID Au nanostructures significantly depends on the total electron dose that certain sections of Au nanostructures experience during deposition. Therefore, one can conclude that the electron beam, primarily used for generating secondary electrons that cause the cracking of precursor molecules and their initial deposition, plays an extra role in FEBID experiments – it cures the pre-deposited material at the same time, likely by additionally breaking the solid carbonaceous fragments of the non-decomposed precursor molecules still present in the deposited material. As gaseous C-containing fragments are pumped out, sections of a relatively higher Au content are left behind. This curing mechanism during FEBID of 2D and 3D structures from the Me_2_-Au-tfac precursor is in essence analogous to the mechanism that governs the deposition from the Me_2_-Au-acac precursor, studied by Wnuk et al. [69–70]. These results show that the Au content in FEBID nanostructures can indeed be significantly increased by setting the proper values of the experimental parameters used for deposition. In general, our experiments indicate that the deposition with higher electron beam doses yields structures of a higher metallic content, in line with the findings of postdeposition Pt curing using high currents and oxygen injection [56]. Looking at a wider perspective, our study may point to a more general strategy for direct realization of exceptionally pure FEBID materials. Moreover, bearing in mind that this deposition technique allows for extremely precise patterning of 2D and 3D structures on the nano- and microscale, these findings may facilitate a widespread usage of FEBID in a range of applications.

### Oxygen-plasma cleaning of FEBID Au nanostructures

As was demonstrated in the previous sections, the experimental results show that, despite significant improvements of the process, a large part of the deposited structures is still carbonaceous material, which is generally undesired. Hence, in the next part of our study of Au nanostructures, we adopted a postdeposition cleaning procedure that would further decrease the C content in the structures. Building up on the work from Miyazoe et al. [76] we here take a similar approach by subjecting the samples to O-plasma, imposing a crucial requirement that the morphology of the structures had to be preserved to a high degree. This requirement is set in order to fully exploit the initial advantage of FEBID over other deposition techniques – direct writing of patterned structures on the nanoscale. Taking this constraint into consideration, it is immediately clear that a substantial removal of carbonaceous material from the deposits represents a major threat to their structural stability and morphology. Thus, the study was focused on finding just the right set of parameters for the O-plasma cleaning procedure that would maximally increase the Au content in the structures, while maintaining the structures’ shape at the same time. In this sense, initial optimization of the Au content during deposition itself seems even more important, because otherwise any postdeposition purification would be significantly limited by the imposed condition of maintaining the structures’ morphology.

**Figure 4:**
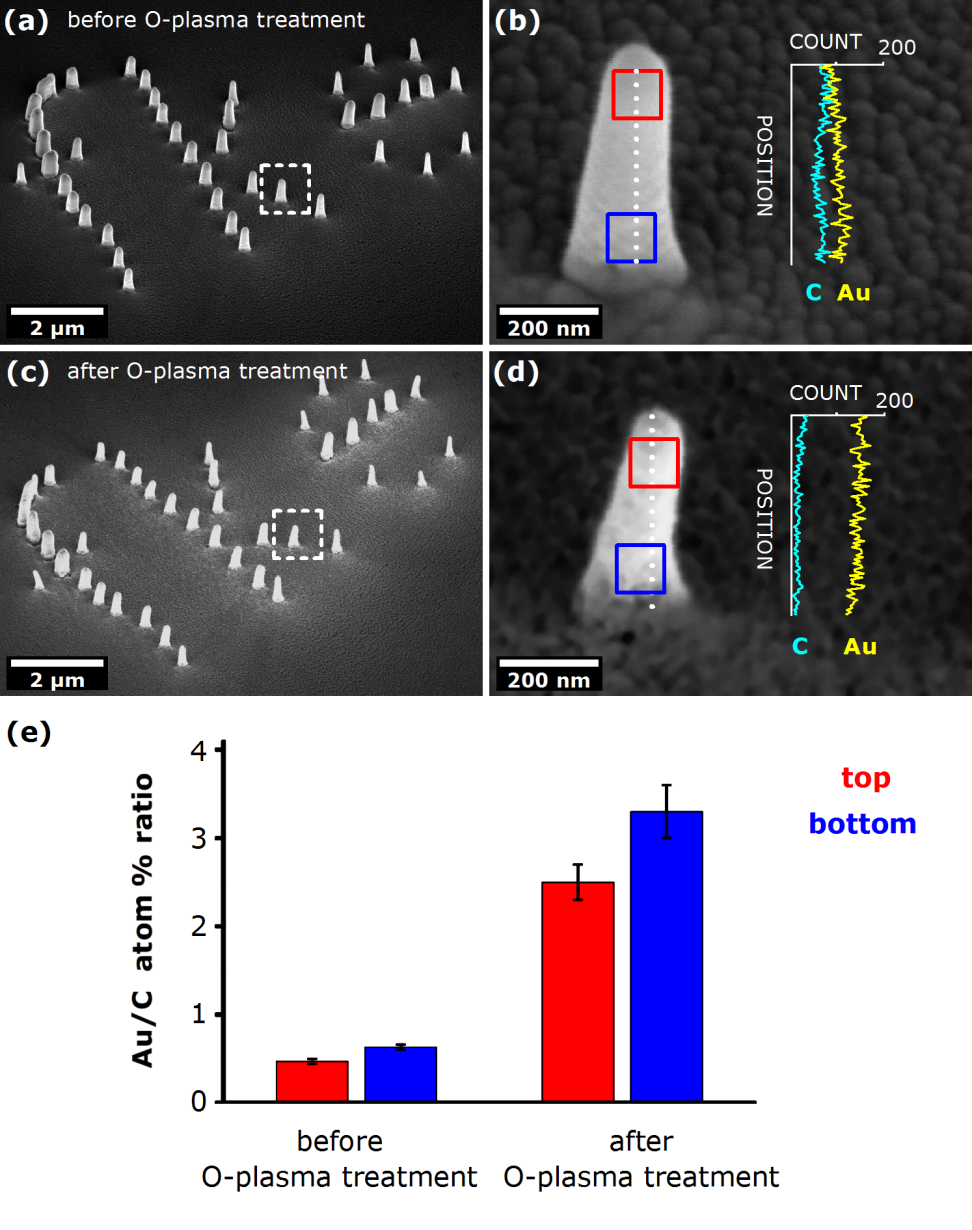
(a) SEM image (tilted view) of the test configuration of patterned pristine Au nanopillars (NPs). Note the variation in the number of neighbouring NPs, i.e., adjacent NPs within 1 μm distance; (b) Close-up view on a single pristine NP from the pattern having 2 neighbouring NPs, with the EDX line profile given in the inset; (c) SEM image (tilted view) of the same configuration after the O-plasma treatment; (d) Close-up view on the same NP as in (b), with the EDX line profile given in the inset. (e) Elemental composition of the top and bottom parts of Au NPs presented in (b,d) acquired before and after O-plasma treatment, expressed as Au/C atom % ratio. The EDX measurements were performed on 100 × 100 nm^2^ areas marked with the red and blue squares in b,d, for a period of 60 s. For detailed EDX data see Table S3 in Supporting Information File 1.

As a test sample, a patterned set of Au NPs was deposited by FEBID (see Figure 4a). We should note that the composition of each NP in this set may slightly differ from the previously discussed stand-alone NPs, since proximity effects are known to come into play when a dense array of FEBID structures is deposited [77–79]. Nevertheless, we think that such a compact configuration might be more similar to the envisaged patterns that will eventually be used in real-life applications, so these NP assemblies may be of greater practical importance.

The NPs were deposited using an acceleration voltage of 3 kV, a beam current of 1 nA, and a deposition time of 60 s. The overall appearance of the complete configuration and a close-up view of a single pristine NP are presented in Figure 4a,b, respectively. To demonstrate the design capabilities of FEBID the NPs form the letters "fke" which is the abbreviated name of the Institut für Festkörperelektronik. It can be seen that the shape of the NPs was not fully uniform across the set: those NPs having more neighbouring NPs appear to have a slightly wider diameter than the more isolated NPs. This is strong evidence of FEBID proximity effects, where deposition of neighbouring NPs leaves some residue on the previously deposited NPs. The EDX study performed on a single pristine NP (conveniently chosen to have two neighbouring NPs, which seems interesting from the application point of view) showed a gradient of Au along the height of the NP (see inset plot in Figure 4b), which was consistent with the results obtained for stand-alone NPs deposited under the same experimental conditions in the first part of the study. Here one has to be careful when deriving conclusions because of a sizeable “halo” deposit present in the surroundings of each NP that may have given a certain background contribution to the EDX line profiles. Such an effect is not pronounced when NPs are deposited far from each other, as described earlier. The EDX probing done on 100 × 100 nm^2^ areas in the top and bottom sections of the NP showed that the NP mostly consisted of a carbonaceous material, having only a minor Au fraction, which was in accordance with the EDX results obtained for stand-alone NPs. These findings seem to be representative for all those NPs in our configuration having two neighbouring NPs. On the other hand, the NPs with three or more neighbouring NPs generally showed slightly higher levels of C content, which can be attributed to more pronounced proximity effects.

Following the initial SEM investigation of pristine Au NPs, the samples were then exposed to O-plasma and examined again using the same SEM parameters. It was immediately evident that some material was indeed removed from the sample, leading to a significant volume reduction (approximately 40–45%) of the O-plasma treated NPs compared to the pristine NPs (see Figure S3, Supporting Information File 1 for detailed analysis). In spite of the loss of material, the stability of individual NPs was largely preserved; consequently, the overall pattern of the NP configuration was conserved as well. The EDX line profiles indicate a prominent increase of the Au content in the NPs: the area-averaged EDX results show increase of the Au fraction, reaching the values of over 70 atom % at the bottom of NPs; at the same time, the C content dropped to below 25 atom %, resulting in a six-fold enhancement of the Au/C atom % ratio due to O-plasma treatment (Figure 4e). Interestingly, this enrichment factor applies almost uniformly to all parts of the NPs, so that the compositional gradient still existed even after the O-plasma treatment. Since the treatment is assumed to have removed carbonaceous species from the surface of the structures, this result implies that the Au gradient in the pristine structures was not a surface-bound feature – it existed throughout the body of the NPs.

**Figure 5:**
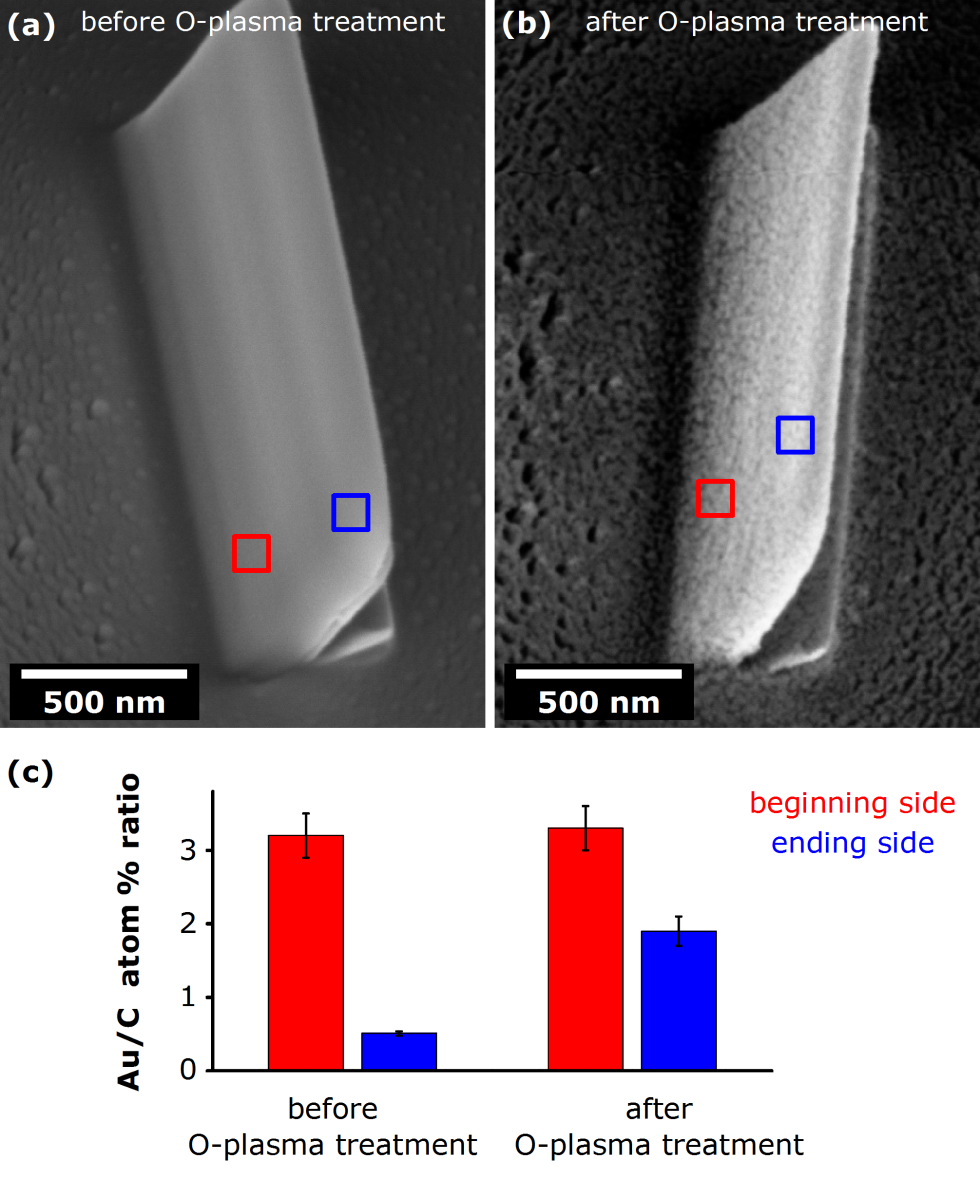
(a) SEM image (tilted view) of a pristine planar FEBID Au nanostructure of a large compositional gradient (see Figure 3d) before O-plasma treatment. (b) SEM image (tilted view) of the same structure after O-plasma treatment. (c) Elemental composition of the “beginning” and “end” of the planar nanostructure, acquired before and after O-plasma treatment. The EDX measurements were performed on 100 × 100 nm^2^ areas for 60 s. For detailed EDX data see Table S4 in Supporting Information File 1.

To confirm that O-plasma cleaning is generally applicable, the same experimental procedure was used on a set of planar samples. We note that, although the underlying cleaning mechanism is assumed the be the same in both planar (2D) and vertical NPs (3D), some differences in the cleaning rate might be expected due to variation in the geometry of exposed surfaces. Consequently, slight modifications in the experimental cleaning protocols between 2D and 3D structures might arise. Again though, a common observation was that, upon O-plasma treatment, the planar nanostructures to a small extent reduced in volume, presumably due to partial removal of carbonaceous material, leaving the original morphology largely preserved. As a rule, the Au fraction increased in all the samples, as revealed by EDX analyses. The enhancement factor tended to be greater for the structures (or their parts) that initially had a lower Au content. A typical example presented in Figure 5 demonstrates this effect quite clearly: a compositionally inhomogeneous pristine planar structure (i.e., structure exhibiting a large Au gradient across the opposite sides) was subjected to O-plasma treatment which resulted in an evident loss of material, rendering its initially smooth surface quite rough. Notably, the shape of the deposit was largely preserved, but more importantly, the EDX analysis showed an increase in Au content throughout the nanostructure. The enhancement factor (EF) was considerably larger on the initially Au-poor, “end” side (EF ≈ 4), while the initially Au-rich “beginning” side saw only a very modest increase (EF ≈ 1.1). We note that, in this way, postdeposition O-plasma treatment may work as a leveller of compositional gradient in inhomogeneous planar FEBID structures.

Furthermore, the increased roughness and SEM-brightness of the nanostructures’ surfaces, seen in Figure 4d and Figure 5b, indicate that the O-plasma cleaning exposed some of the embedded Au nanocrystallites to the exterior. Such purified patterned nanostructures are deemed very suitable for further functionalization, e.g., for immobilization of biomolecules or similar applications where having clean Au surfaces is essential for successful binding of ligands, such as thiols [80]. From that point of view, our cleaning procedure presents a valuable approach for experimental realization of structurally sound, precisely patterned nanostructures of a high Au content, with exposed Au surfaces available for functionalization.

## Conclusion

In summary, we investigated a FEBID experimental parameter subspace, aiming to fabricate 2D and 3D nanostructures with a high Au content, starting from a common metal-organic precursor, Me_2_-Au-tfac. In the first instance, we realized vertical free-standing FEBID Au nanopillars having a compositional gradient along the height of the structure, where the bottom part was systematically Au-richer than the top part, with up to 45 atom % of Au. These results suggest that the electron beam dose sustained during deposition plays a key role in defining the purity of the FEBID Au material. In a similar fashion, a range of planar nanostructures were produced, aiming to identify the optimal set of experimental parameters that will yield deposits of the highest Au content. It was found that, in line with the results obtained for vertical NPs, higher electron beam doses generally leave a purer FEBID Au material, which in the optimal case exceeded 70 atom %. We note that this straightforward approach is feasible with any standard SEM and does not require specialized instrumentation.

The second approach towards higher gold purity is postdeposition purification by oxidation. As molecular oxygen is not reactive enough, oxygen radicals were used. The oxidation was performed in a plasma reactor that is common equipment in most clean rooms. This second purification approach can quickly improve the gold purity of a number of FEBID structures, including larger ones. In this work we developed a process that employs an oxygen-plasma treatment to further increase the purity of the deposited material. It was found that such an approach can significantly increase the Au content in the FEBID nanostructures, while preserving their structural stability. The enhancement factor of Au content was found to be dependent on the initial composition in such a way that the structures (or their parts) that initially had a lower Au fraction experienced a larger boost, levelling the original compositional gradient. We conclude that a combination of optimized deposition followed by a suitable oxygen-plasma cleaning protocol may lead to exceptionally pure, structurally stable, patterned 2D and 3D Au nanostructures. In addition, the oxygen-plasma cleaning might potentially expose the Au surfaces to the exterior, making such freshly processed patterned FEBID Au nanostructures very suitable for prospective surface functionalization, which is highly desired for a range of applications in nanotechnology.

## Experimental

The samples were prepared by FEBID of Au inside a custom-modified Zeiss Leo 1530VP SEM. The microscope was equipped with a home-made gas injection system containing dimethylgold(III)-trifluoroacetylacetonate precursor (Me_2_-Au-tfac, chemical formula C_7_H_10_AuF_3_O_2_). The base pressure in the microscope chamber was around 1.6 × 10^−6^ mbar, while the working pressure during the introduction of precursor gas was maintained at 6.0 × 10^−6^ mbar (except in the case of a large planar structure presented in Figure 1e, where it was 1.2 × 10^−5^ mbar). The deposition took place in a non-reactive environment at room temperature. Customized macros were executed by the SmartSEM user interface to exactly control the electron beam exposure, ensuring complete reproducibility of experimental parameters. When depositing planar structures we made use of a Raith Elphy Plus pattern generator. The samples for SEM analyses were deposited onto ultrasonically cleaned p-type silicon substrates having a thin native oxide layer of around 2 nm, while the samples for TEM investigation were deposited onto a perforated silicon nitride TEM membrane (SPI Supplies). The oxygen-plasma treatment was carried out in a Tepla 100 plasma system using 300 W of power for 180 s, at the gas pressure of 0.9 mbar. At these conditions the main reactive species in the plasma are typically oxygen atoms [81] with an approximate concentration of 10^12^ to 10^13^ atoms/cm^2^ [82].

SEM and EDX characterization was performed in a Zeiss Neon 40EsB cross-beam microscope equipped with an Oxford Instruments EDS 7427 detector, operating at an acceleration voltage of 5 kV and a base pressure of 1.2 × 10^−6^ mbar. The samples were briefly exposed (<2 min) to cleanroom air during the transfer from the Zeiss Leo 1530VP SEM or Tepla 100 plasma system to the Zeiss Neon SEM. TEM characterization was performed in a FEI Tecnai F20 microscope equipped with an EDAX detector and a Fischione 3000 HAADF detector, at an acceleration voltage of 200 kV. The TEM samples were exposed to atmosphere for a few hours during the transfer from the fabrication facility to the TEM lab. AFM investigations were carried out on a Veeco/Bruker Dimension 3000 atomic force microscope.

## Supporting Information

File 1 Additional experimental data.
