# Supplementary material for: Direct writing of gold nanostructures with an electron beam: On the way to pure nanostructures by combining optimized deposition with oxygen-plasma treatment
Source: Beilstein J Nanotechnol. 2017 Nov 29;8:2530–43. doi: 10.3762/bjnano.8.253 (PMC5727840; doi:10.3762/bjnano.8.253)
Supplement: File 1 — Additional experimental data. [file Beilstein_J_Nanotechnol-08-2530-s001.pdf]

Supporting Information

for

Direct-writing of gold nanostructures with an electron-beam: On the way to pure structures by combining optimized deposition with oxygen-plasma treatment

Domagoj Belić[1,2], Mostafa M. Shawrav[1,3], Emmerich Bertagnolli[1] and Heinz D. Wanzenboeck*[1]

Address: [1]Institute of Solid State Electronics, TU Wien, Floragasse 7, 1040 Vienna, Austria, [2]University of Liverpool, Department of Chemistry, Crown Street, Liverpool L69 7ZD, United Kingdom and [3]Institute of Sensors & Actuator System, TU Wien, Gusshausstrasse 27–29, 1040 Vienna, Austria

Email: Heinz D. Wanzenboeck - Heinz.wanzenboeck@tuwien.ac.at

* Corresponding author

Additional experimental data

Please note: all EDX data presented in this document are given for descriptive purposes - to show a general trend, rather than to provide the exact values to the first decimal point.

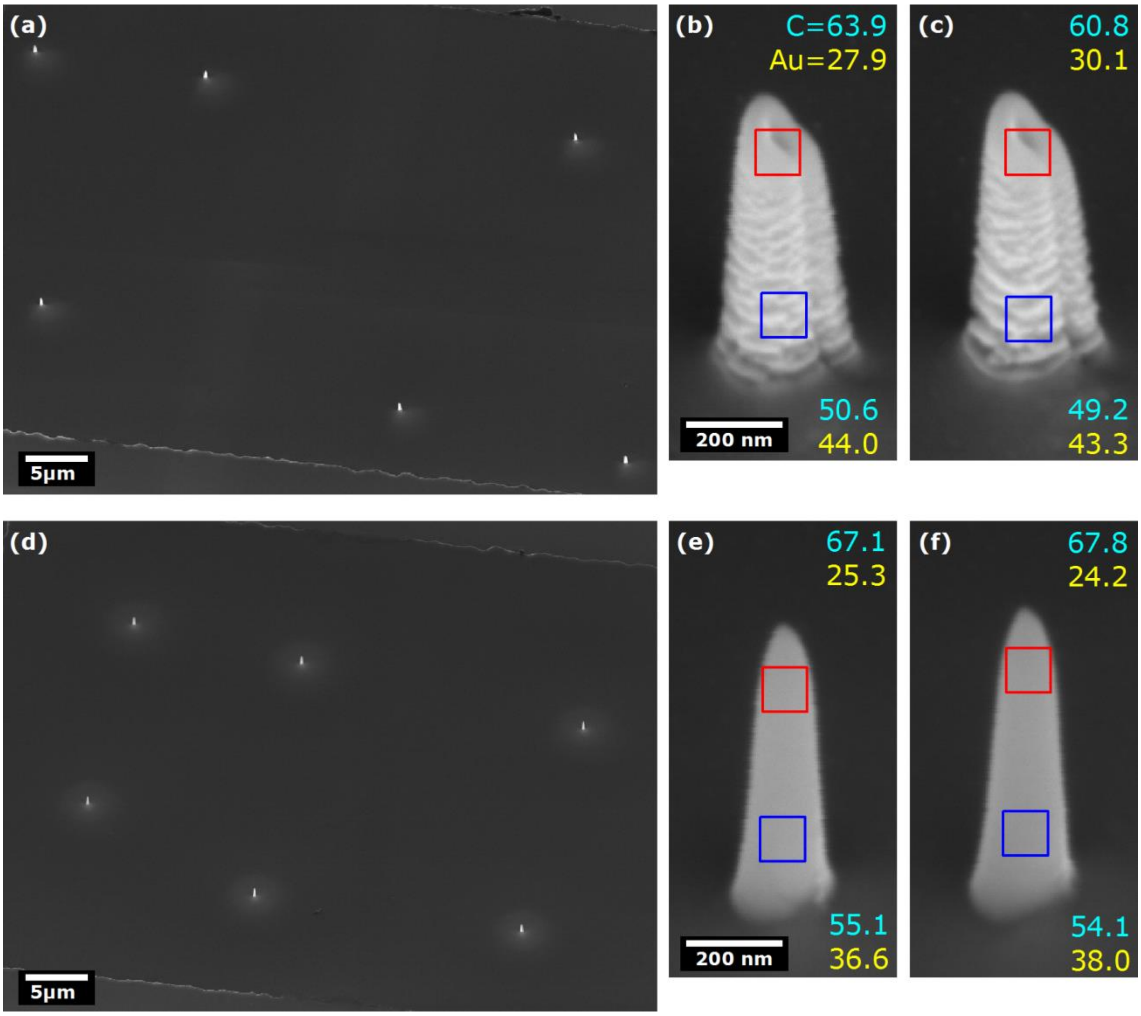

Figure S1: SEM images and EDX data (carbon content indicated blue, gold indicated yellow) for two sets of samples of FEBID Au nanopillars (NPs), showing very similar morphological and compositional profiles within each set of samples. (a) Low magnification SEM image of a set of Au NPs fabricated at HT=1 kV, I=1.0 nA and t=60 s. (b – c) High magnification SEM images of 2 NPs from this set. EDX atomic percentage for carbon and gold is indicated on the images, based on 60s acquisition from 100 × 100nm^2 areas in the top and bottom part of the NPs. (d) Low magnification SEM image of a set of Au NPs fabricated at HT=10 kV, I=1.0 nA and t=60 s. (e – f) High magnification SEM images of 2 NPs from that set and the corresponding EDX data.

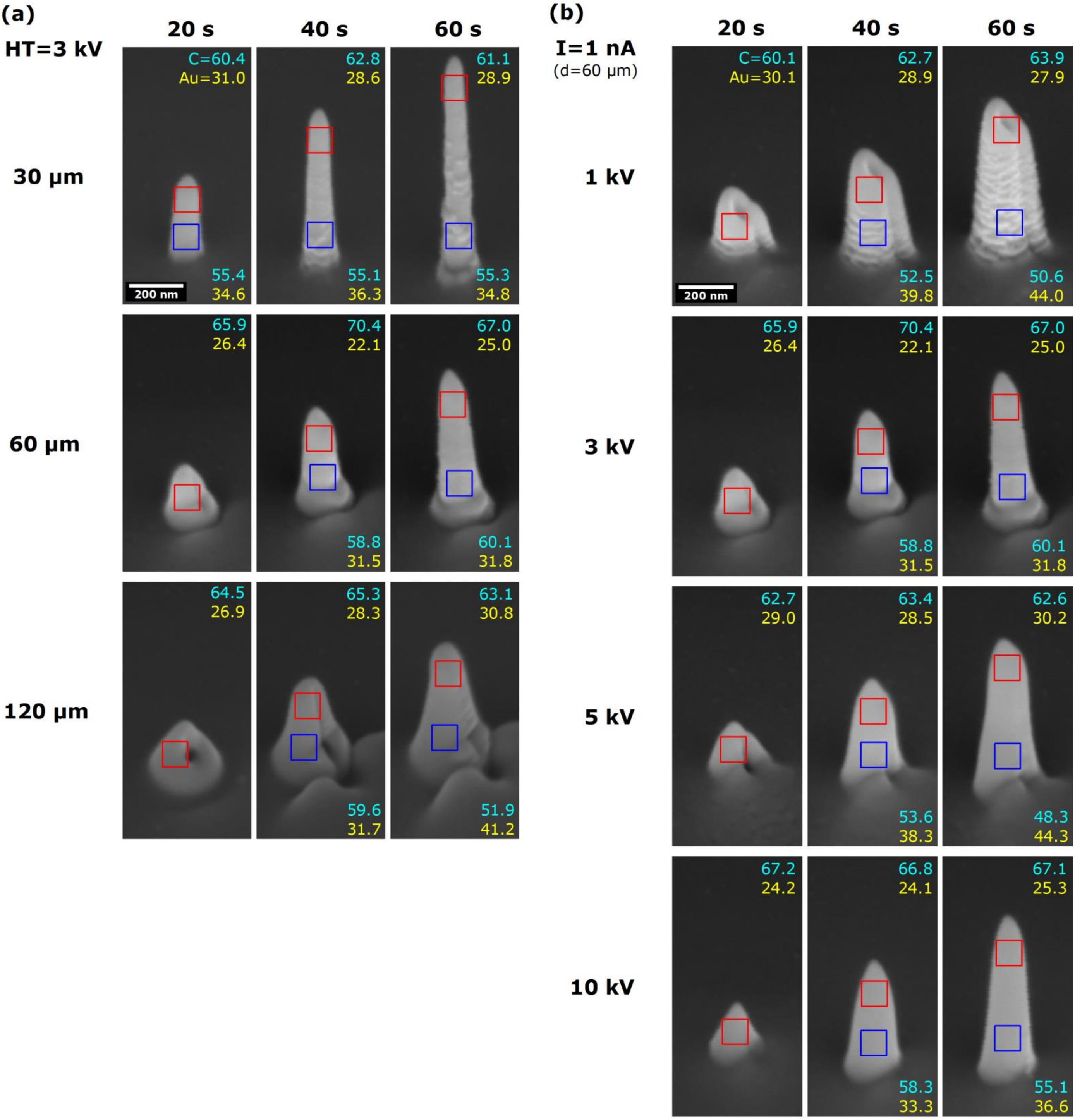

Figure S2: (a) SEM images and EDX analysis of representative FEBID Au NPs deposited at a constant of HT=3 kV, for various apertures (d=30 µm, 60 µm, and 120 µm) and deposition times (t=20 s, 40 s, and 60 s). Note that the system was optimized to maintain the constant electron beam current of I=1 nA when the 60 µm aperture was used. The scale bar is applicable to all images. (b) SEM images and EDX analysis of representative FEBID Au NPs deposited at a constant current of 1 nA (i.e. using the 60 µm aperture) for various acceleration voltage values (HT= 1 kV, 3 kV, 5 kV, and 10 kV) and deposition times (t=20 s, 40 s, and 60 s).

Table S1: EDX compositional data acquired on 100 × 100 nm^2 areas in the top and bottom sections of the gold nanopillars presented in Figure 2 (a – d) in the main document.

Sample (Figure)	Gold [atom %]	Carbon [atom %]	Oxygen [atom %]	Gold - relative difference bottom/top [%]
1 kV top (2b)	(30.1 ± 0.3)	(61 ± 2)	(9.1 ± 0.9)	+ 43.9
1 kV bottom (2b)	(43.3 ± 0.2)	(49 ± 2)	(7.5 ± 0.9)	
3 kV top (2c)	(23.8 ± 0.3)	(69 ± 2)	(7.1 ± 0.8)	+ 25.2
3 kV bottom (2c)	(29.8 ± 0.2)	(62 ± 2)	(8.0 ± 0.9)	
5 kV top (2d)	(30.2 ± 0.3)	(63 ± 2)	(7.2 ± 0.9)	+ 46.7
5 kV bottom (2d)	(44.3 ± 0.2)	(48 ± 2)	(7.4 ± 0.9)	
10 kV top (2e)	(24.2 ± 0.3)	(68 ± 2)	(8.0 ± 0.9)	+ 57.0
10 kV bottom (2e)	(38.0 ± 0.2)	(54 ± 2)	(8 ± 1)	

Table S2: EDX compositional data acquired on 100 × 100 nm^2 areas at the "beginning" and "ending" side of the planar gold nanostructures presented in Figure 3 (c – f) in the main document.

Sample (Figure)	Gold [atom %]	Carbon [atom %]	Oxygen [atom %]	Gold - relative difference beginning/ending side [%]
HT=1kV, PPS=3nm, DT=2.048ms, beginning side (3a)	(32.1 ± 0.3)	(61 ± 2)	(9.2 ± 0.9)	+ 17.6
HT=1kV, PPS=3nm, DT=2.048ms, ending side (3a)	(27.3 ± 0.2)	(64 ± 2)	(8.7 ± 0.7)	
HT=1kV, PPS=3nm, DT=0.2048ms, beginning side (3b)	(72.5 ± 0.3)	(23 ± 2)	(4 ± 1)	+ 230.9
HT=1kV, PPS=3nm, DT=0.2048ms, ending side (3b)	(31.4 ± 0.2)	(61 ± 2)	(7.8 ± 0.8)	
HT=5kV, PPS=3nm, DT=2.048ms, beginning side (3c)	(31.3 ± 0.2)	(61 ± 2)	(7.6 ± 0.8)	+ 13.8
HT=5kV, PPS=3nm, DT=2.048ms, ending side (3c)	(27.5 ± 0.1)	(64 ± 2)	(9.1 ± 0.7)	
HT=5kV, PPS=3nm, DT=0.2048ms, beginning side (3d)	(68.7 ± 0.3)	(26 ± 2)	(5.1 ± 0.9)	+ 233.7
HT=5kV, PPS=3nm, DT=0.2048ms, ending side (3d)	(29.4 ± 0.2)	(63 ± 2)	(8.0 ± 0.7)	

Table S3: EDX compositional data acquired on 100 × 100 nm^2 areas in the top and bottom sections of the gold nanopillar (before and after O-plasma treatment) presented in Figure 4 (b, d) in the main document.

Sample (Figure)	Gold [atom %]	Carbon [atom %]	Oxygen [atom %]	Gold – relative change after O-plasma [%]
top, before O-plasma (4b)	(29.9 ± 0.2)	(63 ± 2)	(7.3 ± 0.8)	
bottom, before O-plasma(4b)	(35.9 ± 0.2)	(57 ± 2)	(7.5 ± 0.8)	
top, after O-plasma (4d)	(67.2 ± 0.2)	(27 ± 2)	(6 ± 1)	+ 224.7
bottom, after O-plasma (4d)	(71.7 ± 0.2)	(22 ± 2)	(6 ± 1)	+ 199.7

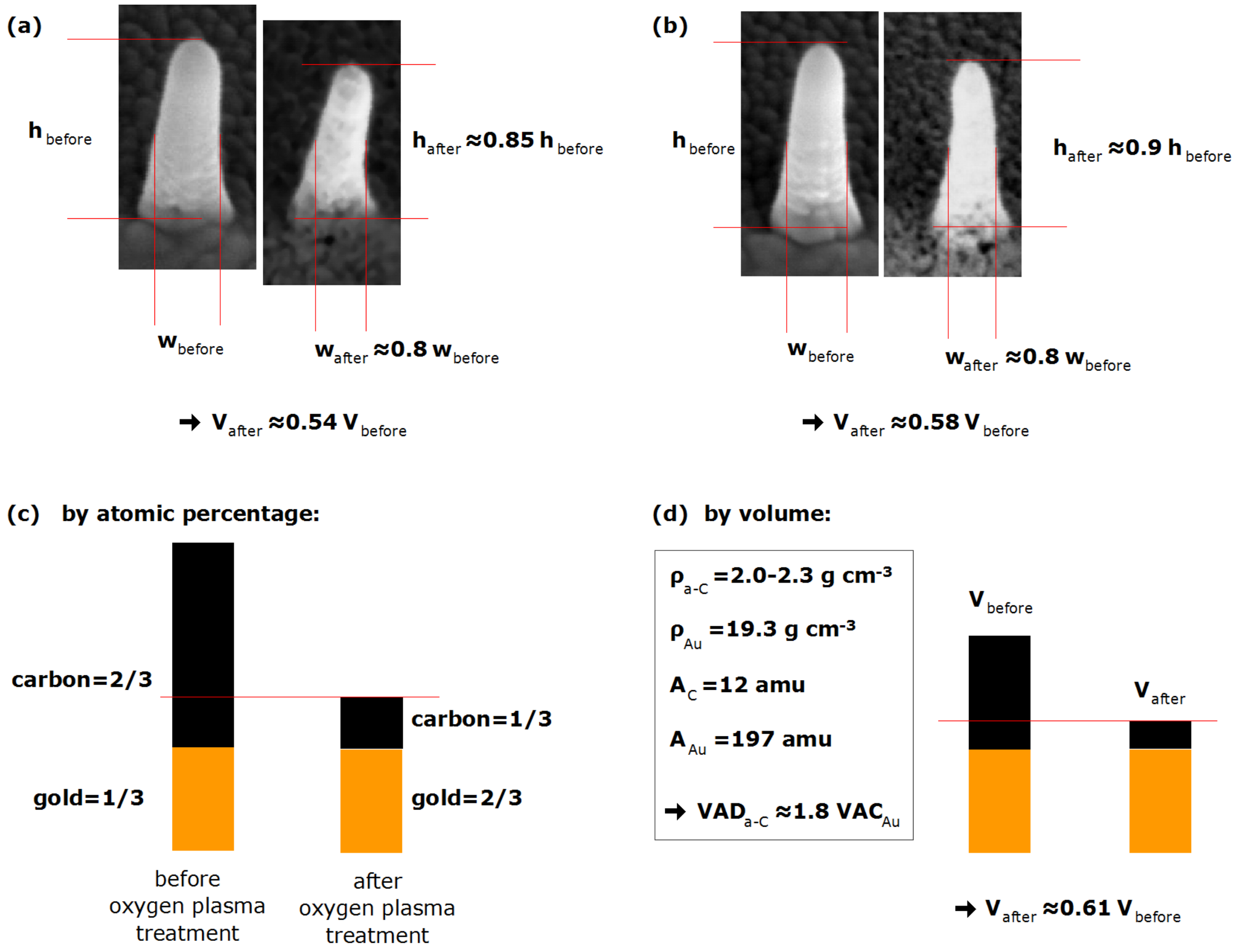

Figure S3: Quantitative analysis of NP volume reduction after oxygen plasma cleaning. (a, b) High magnification SEM image analysis of 2 NPs before and after treatment reveals a loss of approximately 40-45% of material. (c) Our EDX measurements show that the pristine NPs contain roughly 2/3 carbonaceous material and 1/3 gold (note: by atomic percent), while purified NPs contain roughly 1/3 carbon and 2/3 gold). Assuming no gold was removed by oxygen plasma cleaning, it means that ¾ of initial carbonaceous material (by number of atoms) must have been removed during the process. (d) When the volumetric atomic densities (VAD) of amorphous carbon and (nano)crystalline gold are taken into account (where ρ is common mass density and A is atomic mass of the corresponding species; bulk values though), one might

calculate the expected loss of volume. In our case, based on the initial and final composition of NPs, the calculation yields a volume reduction of roughly 39%, which closely matches the observed loss of volume.

Table S4: EDX compositional data acquired on 100 × 100 nm^2 areas in the top and bottom sections of the planar nanostructure (before and after O-plasma treatment) presented in Figure 5 (a, b) in the main document.

Sample (Figure)	Gold [atom %]	Carbon [atom %]	Oxygen [atom %]	Gold – relative change after O-plasma [%]
beginning side, before O-plasma (5a)	(72.5 ± 0.3)	(23 ± 2)	(4 ± 1)	
ending side, before O-plasma (5a)	(31.4 ± 0.2)	(61 ± 2)	(7.8 ± 0.8)	
beginning side, after O-plasma (5b)	(72.9 ± 0.3)	(22 ± 2)	(5 ± 1)	+ 0.6
ending side, after O-plasma (5b)	(56.5 ± 0.3)	(30 ± 2)	(13 ± 1)	+ 79.9
